# Adaptation processes in *Halomicronema hongdechloris*, an example of the light-induced optimization of the photosynthetic apparatus on hierarchical time scales

**DOI:** 10.3389/fpls.2024.1359195

**Published:** 2024-07-10

**Authors:** Franz-Josef Schmitt, Thomas Friedrich

**Affiliations:** ^1^ Department of Physics, Martin-Luther-Universität Halle-Wittenberg, Halle, Germany; ^2^ Department of Bioenergetics, Technische Universität Berlin, Institute of Chemistry PC 14, Berlin, Germany

**Keywords:** *H. hongdechloris*, chlorophyll *f*, far-red light photoacclimation, electron tunneling, uphill energy transfer, charge separation, hierarchical time scales, light-induced optimization

## Abstract

Oxygenic photosynthesis in *Halomicronema hongdechloris*, one of a series of cyanobacteria producing red-shifted Chl *f*, is adapted to varying light conditions by a range of diverse processes acting over largely different time scales. Acclimation to far-red light (FRL) above 700 nm over several days is mirrored by reversible changes in the Chl *f* content. In several cyanobacteria that undergo FRL photoacclimation, Chl *d* and Chl *f* are directly involved in excitation energy transfer in the antenna system, form the primary donor in photosystem I (PSI), and are also involved in electron transfer within photosystem II (PSII), most probably at the Chl_D1_ position, with efficient charge transfer happening with comparable kinetics to reaction centers containing Chl *a*. In *H. hongdechloris*, the formation of Chl *f* under FRL comes along with slow adaptive proteomic shifts like the rebuilding of the D1 complex on the time scale of days. On shorter time scales, much faster adaptation mechanisms exist involving the phycobilisomes (PBSs), which mainly contain allophycocyanin upon adaptation to FRL. Short illumination with white, blue, or red light leads to reactive oxygen species-driven mobilization of the PBSs on the time scale of seconds, in effect recoupling the PBSs with Chl *f*-containing PSII to re-establish efficient excitation energy transfer within minutes. In summary, *H. hongdechloris* reorganizes PSII to act as a molecular heat pump lifting excited states from Chl *f* to Chl *a* on the picosecond time scale in combination with a light-driven PBS reorganization acting on the time scale of seconds to minutes depending on the actual light conditions. Thus, structure–function relationships in photosynthetic energy and electron transport in *H. hongdechloris* including long-term adaptation processes cover 10^−12^ to 10^6^ seconds, i.e., 18 orders of magnitude in time.

## 1 Introduction

The reaction centers in oxygenic photosynthesis exhibit a notable uniformity across photosynthetic organisms, characterized by structurally analogous components in photosystem I (PSI) and photosystem II (PSII). However, this homogeneity contrasts starkly with the diversity and rapid adaptability of light-harvesting systems (for a recent review, see Lokstein et al., 2021). These systems exhibit rapid adaptation mechanisms, including light-induced dynamics, e.g., state transitions between PSII and PSI (Minagawa, 2011) or light-induced or non-photochemical quenching that can occur within seconds. The variety of light-induced quenching processes often involves carotenoids, e.g., in the xanthophyll cycle, in which photochemistry and photoprotection are balanced depending on light conditions (Short et al., 2023). The activation and recovery of the Orange Carotenoid Protein mediating the non-photochemical quenching of phycobilisome (PBS) fluorescence is another prominent example (Maksimov et al., 2013; Kirilovsky et al., 2014, 2014, 2015, 2016; Moldenhauer et al., 2018). These mechanisms are crucial for optimizing photosynthesis while protecting the organisms from the effects of fluctuating light levels in the natural environments (Short et al., 2023).

Regulatory processes occur hierarchically on different time scales. It is known that the chlorophyll content of antennae is modulated by the activation of photoreceptors such as phytochromes, cryptochromes, and phototropins (Quail, 2010; Su et al., 2017; Kong and Okajima, 2018; Kreslavski et al., 2018). In particular, phytochromes orchestrate a wide range of physiological responses, from seed germination to flowering and fruiting (Carvalho et al., 2011; Su et al., 2017; Kreslavski et al., 2018). In addition, this system exerts regulatory control over the plant’s metabolic response to various environmental stresses such as temperature, salinity, drought, and UV radiation (Franklin and Whitelam, 2007; Carvalho et al., 2011; 2016; Kreslavski et al., 2013a, 2013b; 2016, 2017). Phytochromes modulate enzyme activities that control metabolic processes, especially the biosynthesis of small molecule antioxidants and photosynthetic pigments, as well as the expression levels of genes involved in cellular signaling (Kreslavski et al., 2016, 2018; Schmitt et al., 2014a; Schmitt and Allakhverdiev, 2017).

The adaptive responses of various species to light conditions, particularly by modification of the association between light-harvesting complexes (LHCs) and PSII and/or PSI, are remarkably diverse (Ueno et al., 2019). These adaptations, first observed by Murata in 1969 in red algae containing PBSs (Murata, 1969), facilitate the redistribution of excitation energy between photosystems in cyanobacteria via the mobility of the PBSs on time scales ranging from seconds to days (Sherman et al., 1998; Kirilovsky et al., 2014; Schmitt et al., 2020). Adaptive mechanisms redistributing light energy between PSII and PSI extend to higher plants, as has been extensively reviewed in the literature (Mullineaux et al., 1997; Mullineaux and Emlyn-Jones, 2005; Ruban and Johnson, 2009; Papageorgiou and Govindjee, 2011). The mechanisms driving these light-induced adaptations are complex and involve reactive oxygen species (ROS) and activation of photoreceptors and histidine kinases that modulate gene expression through phosphorylation/dephosphorylation (Minagawa, 2011; Schmitt et al., 2014a; Schmit et al., 2014b; Schmitt and Allakhverdiev, 2017; Schmitt et al., 2020).

The diffusional properties of phycobiliprotein (PBP) antenna complexes represent an evolutionary adaptation to environmental stress and/or changing light conditions, possibly mediated by charge variations on the protein surface, ultimately affecting the amount of light energy harnessed for photochemistry (Schreiber, 1980; Schmitt et al., 2006; Schmitt, 2010; Schmitt, 2011; Schmitt et al., 2020).

Complementary chromatic adaptation (CCA), initially documented in 1903 (Gaidukov, 1903), is a process in which cyanobacteria alter their pigment composition in response to the spectrum of ambient light, with the goal of optimizing light harvesting by synthesizing specific PBS components. Red light (RL) stimulates an increase in the production of phycocyanin (PC), which efficiently absorbs photons in the 550–650-nm range, while green light (GL) prompts increased synthesis of phycoerythrin (PE), which absorbs light between 500 and 600 nm. Kehoe and Grossman’s extensive review provides a detailed historical overview of CCA in cyanobacteria (Kehoe and Grossman, 1994).

Subsequent research in photobiology using mutagenesis has further unraveled the mechanistic aspects of CCA. Hirose et al. (2010) identified a cyanobacteriochrome (CcaS) and its associated response regulator as key players in regulating PE synthesis in *Nostoc punctiforme*. The GAF domain of CcaS alternates between RL-absorbing (Pr) and GL-absorbing (Pg) states, modulating the activity of the histidine kinase module, which in turn regulates the transcription of genes responsible for PE biosynthesis. These findings highlight the functional similarities between cyanobacteriochromes in cyanobacteria and phytochromes in higher plants, both of which orchestrate pigment composition adaptations in response to environmental light conditions (Kreslavski et al., 2013a; 2013b; 2017; 2018).

Beyond PBSs, light-induced pigment alterations are also evident in PSI and PSII across photosynthetic organisms encompassing cyanobacteria, green algae, and higher plants (Hirose et al., 2010; Kreslavski et al., 2013a; 2013b; 2017;Schmit et al., 2014b; Schmitt and Allakhverdiev, 2017). Recent investigations of the structural adaptations in certain cyanobacteria such as *Halomicronema hongdechloris* have revealed their ability to acclimate to far-red light (700–800 nm) through a process known as far-red light photoacclimation (FaRLiP). This adaptation involves significant modifications to the photosystems’ subunit composition and pigment arrangement, notably involving the incorporation of Chl *d* and Chl *f*. Chl *f* is the most red-shifted chlorophyll known to date and is derived from Chl *a* by the addition of a formyl group at the C2 position, classifying it as [2-formyl]-Chl *a* (Chen et al., 2010; Willows et al., 2013; Lindsey et al., 2015). Selective culturing under far-red light (FRL) illumination demonstrated that *H. hongdechloris* produces Chl *f* during FaRLiP (Chen et al., 2012). Such a change enables the organism to extend the spectrum of light absorption beyond the conventional red limit of oxygenic photosynthesis. A detailed understanding of the mechanisms behind FaRLiP will not only enhance our understanding of photosynthetic diversity and adaptability under spectrally extreme light conditions but also advance the bioengineering of other photosynthetic organisms to utilize far-red light (Gisriel, 2024).

Ongoing debates focus on the primary and secondary electron donors within the PSII reaction center (RC) of *H. hongdechloris*, particularly regarding the chemical nature and structural positioning of the chlorins serving as the primary donor (PD) and secondary donor. Recent investigations on *Chroococcidiopsis thermalis* suggested a Chl *d* as PD at the Chl_D1_ position, with likely a red-shifted Chl *a* as a secondary donor at the P_D1_ position (Nürnberg et al., 2018; Judd et al., 2020; Zamzam et al., 2020). A recent cryogenic electron-microscopy (cryo-EM) study of far-red light-adapted PSII from *Synechococcus* sp. PCC 7335 at 2.25 Å resolution also identified a Chl *d* molecule at the Chl_D1_ position, with Chl *a* preserved at the P_D1_ position even under FRL illumination (Gisriel et al., 2022; MacGregor-Chatwin et al., 2022; Gisriel, 2024), akin to our findings for *H. hongdechloris*, which has either Chl *d* or Chl *f* at Chl_D1_ and probably exhibits a similar architecture (Schmitt et al., 2024). According to Gisriel et al., four additional Chl *f* molecules are found in the core antenna contributing to FRL absorption in *Synechococcus* sp. PCC 7335 after FaRLiP (Gisriel et al., 2022; Gisriel, 2024).

Silori et al. (2023) utilized two-dimensional electronic spectroscopy to show that charge separation in FRL-adapted PSII involves rapid interaction between Chl_D1_ and P_D1_, occurring within approximately 3 ps. This process notably bypasses Pheo_D1_, which traditionally is considered to be the primary electron acceptor (Silori et al., 2023). The charge separation efficiency is surprisingly high after FaRLiP, which emphasizes the intricate designs developed by these organisms to maintain the high photochemical efficiency of both photosystems (Tros et al., 2021; Mascoli et al., 2022). Of note, the kinetics of excitation energy transfer (EET) from red-shifted chlorophylls within the antenna system to the reaction centers suggest a transient energy storage mechanism followed by an uphill EET upon FaRLiP, which finally leads to charge separation at the RC (Trissl, 1993; Croce et al., 2000; Gobets and van Grondelle, 2001; Jennings et al., 2003; Allakhverdiev et al., 2016).

Viola et al. investigated *Acaryochloris marina*, which predominantly contains Chl *d*, and compared it to the FaRLiP-competent species *C. thermalis* (Gisriel et al., 2022; Viola et al., 2022; Gisriel, 2024). The charge separation dynamics of both organisms was compared with reaction centers harboring Chl *a*. The findings revealed that all three PSII types have similar turnover numbers, indicating that the adaptation to far-red light does not compromise the catalytic activity of PSII. However, the study identified significant differences in how these systems deal with energy conversion and photodamage. Chl *d*-PSII, while efficient in charge separation, showed a propensity for increased singlet oxygen production under high light conditions, leading to higher photodamage susceptibility compared to PSII containing Chl *a*. Conversely, Chl *f*-PSII demonstrated a balance between efficient light usage and resilience to photodamage, suggesting an optimization that favors damage avoidance without significantly compromising efficiency (Viola et al., 2022). Furthermore, the acceptor-side energies in Chl *f*-PSII are tuned to avoid harmful back reactions. This is explained by differences in the redox tuning of the electron transfer cofactors Pheo_D1_ and Q_A_ and the number and layout of the chlorophylls that share the excitation energy with the primary electron donor. In *A. marina* (harboring Chl *d*-PSII) and organisms that undergo FaRLiP, the acclimation to far-red light has developed into two distinct directions, each appropriate for its specific environment but with different functional penalties (Viola et al., 2022). This agrees with the fact that *A. marina* shares the environment with the Chl *a*-containing phototropic obligate symbiont *Prochloron didemni*, which absorbs white light up to 680 nm (Bibby et al., 2003; Chen et al., 2005; Petrásek et al., 2005; Itoh et al., 2007; Mielke et al., 2011; Schlodder et al., 2007; Theiss et al., 2011) Due to this competition, *A. marina* continuously faces far-red enriched illumination and is evolutionarily adapted to FRL. FaRLiP, in contrast, is a temporary phenomenon that needs to balance efficient far-red light-harvesting and subsequent charge separation on the one hand and photodamage due to sudden changes in the illumination on the other. Our review shows that *H. hongdechloris* as a paradigmatic FaRLiP-capable species employs a series of adaptation mechanisms, including high PBS mobility, and the possibility to form different types of PSII that contain either Chl *a* only or Chl *d* or Chl *f*, which are better suited to absorb FRL. As will be described below, the latter PSII types are not energetically coupled with PBSs after FRL acclimation (Schmitt et al., 2020). Due to varying light conditions, *H. hongdechloris* needs strong protection against ROS, which is provided by enriched carotenoid content after FaRLiP (Chen et al., 2012; Schmitt et al., 2019; 2020; 2024).

Former studies of cyanobacterial core complexes and eukaryotic PSI/LHC systems have revealed that red-shifted chlorophylls in antenna systems function as energy traps, decelerating the energy flow to RCs and facilitating a gradual, energetically uphill EET to the special pair for charge separation (Croce et al., 2000; Jennings et al., 2003; Gobets and van Grondelle, 2001; Trissl, 1993). This thermally activated energy transfer aligns with the Arrhenius–Eyring theory, as observed in the PSI/LHC system in maize thylakoids (Croce et al., 2000; Jennings et al., 2003). Analogous phenomena have been documented in the *Ostreobium* algal species, highlighting the necessity for balanced operation of both photosystems under FRL conditions to ensure efficient carbon fixation (Wilhelm and Jakob, 2006). Moreover, anti-Stokes fluorescence in chloroplasts, characterized by upshifts exceeding 100 nm upon excitation with 800-nm light, exemplifies uphill energy transfer (Hasegawa et al., 2011). The strong temperature dependence of this thermal uphill energy transfer also allows local temperature determination (Hasegawa et al., 2011; Schmitt et al., 2019).

Recent research has elucidated that red-shifted Chl *f*, possessing an absorption peak nearing 800 nm, plays a pivotal role in channeling energy to the primary donor within the PSI of FRL-adapted *Synechococcus* sp. PCC 7335 cells. The PD in PSI is likely a Chl *a*/Chl *a*′ heterodimer, exhibiting an absorption peak at 704 nm (Kurashov et al., 2019). Comparable mechanisms were postulated for the PSI of *H. hongdechloris* (Kato et al., 2020). The uphill energy transfer in *H. hongdechloris*, which bridges an energy gap of approximately 13 kJ/mol, is enhanced by an entropy gain. This is due to a maximum Chl *f*/Chl *a* ratio of 1:8 achieved under FRL illumination. This creates a situation in which a few Chl *f* molecules are energetically coupled to a larger pool of Chl *a* molecules. In turn, the free energy gap during the uphill energy transfer process is diminished (Schmitt et al., 2019). This entropy effect significantly accelerates the endothermal EET, given that the exciton can migrate into a highly degenerate ensemble of coupled and blue-shifted acceptor molecules after light absorption.

The role of Chl *f* in PSI has been widely addressed. A detailed review by Elias et al. uncovered several specific findings related to the structural and functional adaptations of PSI to FRL in various cyanobacteria, highlighting the complex interplay of genetic, structural, and environmental factors that enable these organisms to thrive under FRL conditions (Elias et al., 2024). A comparison of the structures of FRL-PSI from *Fischerella thermalis* PCC 7521 and *Synechococcus* sp. PCC 7335, together with reanalyzed data from *H. hongdechloris*, provided a clearer understanding of FRL-PSI. It was noted that Chl *f* sites, which are conserved across these organisms, are crucial for uphill energy transfer to reaction center chlorophylls, as supported by spectroscopic analyses. The presence of Chl *f* at specific sites of FRL-PSI complexes suggests a mechanism based on nuanced binding affinity, in which not all sites exclusively bind Chl *f*, with some lacking specificity for Chl *f*, allowing Chl *a* to bind under different conditions. This finding challenges the concept of mutually exclusive site occupancy and emphasizes the adaptive flexibility of photosystems in response to environmental light conditions. The study of Elias et al. also highlighted minor species-specific variations in Chl *f* occupancy within FRL-PSI structures, suggesting some genetic diversity in FRL acclimation among different cyanobacteria. This species specificity in Chl *f* placement within PSI underscores the evolutionary adaptability of cyanobacteria to their light environment (Elias et al., 2024).

These findings collectively enhance our understanding of the structural adaptations in cyanobacterial photosystems that facilitate efficient energy capture and conversion under FRL conditions and shed light on the mechanisms supporting photosynthetic efficiency and adaptability in changing light environments (Elias et al., 2024). A study by MacGregor-Chatwin et al. showed that large-scale alterations in the distribution of PSI induced by FaRLiP in *C. thermalis* PCC 7203, *Synechococcus* sp. PCC 7335, and *Chlorogloeopsis fritschii* PCC 9212 show strong cell-to-cell variability of the FaRLiP response, demonstrating how FRL impacts the organization and function of photosynthetic components at the cellular level (MacGregor-Chatwin et al., 2022).

Our studies of *H. hongdechloris* revealed that this species employs several of the mentioned adaptation mechanisms under varying light conditions. These include CCA through i) the synthesis of FRL-absorbing Chl *f* in both the antenna and RC of PSI and PSII, ii) chromophore shifts in the PBS antenna complexes to far-red-absorbing allophycocyanine (APC), and iii) rapid state transitions of the PBSs between PSII complexes with varied pigment compositions (Schmitt et al., 2019; Ho et al., 2020; Schmitt et al., 2020: Schmitt et al., 2024). In FRL-adapted cells re-exposed to white light (WL), PBS transitions from mainly Chl *a*-containing PSII to functionally active Chl *f*-containing PSII were observed, a process that facilitates efficient energy transfer from PBSs to all RCs (Schmitt et al., 2020). These findings show that redistribution and remodeling of the PSII pigment composition are general mechanisms that operate on different time scales and appear as variations in the protein dynamics that are coupled to proteomic shifts.

We focused on rationalizing the kinetics of the time-resolved fluorescence observed in whole cells and developed a kinetic model to understand the fluorescence emission based on the currently accepted chromophore composition of the FRL-adapted RC of PSII according to Gisriel et al. (2022) and Gisriel (2024). *H. hongdechloris* demonstrates a sophisticated hierarchy of mechanisms for regulating energy and electron transfer in PSII (Chen et al., 2005; Petrásek et al., 2005; Schlodder et al., 2007; Theiss et al., 2011 ). Chl *f* plays a critical role in energy and electron transfer processes within PSII, leading to intriguing adaptation mechanisms on time scales of up to 10 days. Key proteins affected include PBSs, which change their composition depending on light conditions degenerating to proteins predominantly containing APC and genetically modified forms of red-absorbing APCs formed during FaRLiP (Li et al., 2016). In addition, enzymes involved in chlorophyll synthesis such as Chl *f* synthase, and regulatory proteins like phytochromes, which act as light sensors and initiate the CCA response, are integral to the proteomic changes observed in a large variety of FaRLiP-capable cyanobacteria (Gan et al., 2014; 2015; Ho et al., 2016; Chen et al., 2019).

Research on *H. hongdechloris* has shown that Chl *f* is not initially present in the D1 protein complexes but may be synthesized in response to FRL exposure. While Chl *a* is the predominant Chl *f*orm under white light conditions, exposure to FRL activates/upregulates specific enzymes, such as the recently identified Chl *f* synthase (encoded by the *psbA*4 gene, also termed *chlF*), which catalyzes the conversion of Chl *a* or its immediate precursor chlorophyllide *a* to Chl *f*. This enzymatic transformation involves a photo-oxidoreductive reaction that is particularly dependent on light of wavelengths longer than 700 nm (Ho et al., 2016). The Chl *f* formed in turn may then be incorporated into the D1 protein complexes instead of Chl *a* molecules. The incorporation process is tightly regulated by the FaRLiP gene cluster, which ensures precise tuning of the photosynthetic apparatus to the available light spectrum, thus enhancing the overall efficiency and adaptability of photosynthesis in *H. hongdechloris* (Ho et al., 2016).

Chl *f* synthase in *H. hongdechloris* is a remarkable example of how a single enzyme can profoundly influence the organism’s proteomic landscape, particularly in its photosynthetic apparatus. This enzyme is instrumental in producing a variant of the D1 protein, a key component of the PSII reaction center, which is adapted to efficiently utilize FRL. The D1 protein is responsible for driving the electron transport chain. Typically, in most cyanobacteria and plants, the D1 protein is configured to function optimally with Chl *a*. However, the generation of a Chl *f*-compatible D1 protein analog in *H. hongdechloris* represents a significant proteomic alteration. This modified D1 protein leads to i) altered light absorption properties since binding Chl *f* instead of Chl *a* changes the light-harvesting characteristics of PSII, thereby enabling efficient utilization of FRL; ii) an impact on electron transport since the electron transport properties of Chl *f* differ from those of Chl *a*, necessitating tuning of electron transfer within the new spectral context; iii) a requirement for coordinated changes in other proteins, as the presence of Chl *f*-bound D1 protein likely invokes adjustments of other PSII components and the associated light-harvesting complexes for optimal functionality; and iv) triggering of further proteomic adaptations to harmonize the entire photosynthetic machinery with the new light environment (Ho et al., 2016; Chen et al., 2019).

To this end, *H. hongdechloris* upon FRL acclimation utilizes a molecular heat pump driven by entropy and supported by electron tunneling, which is known as a key mechanism involved in water splitting, in conjunction with light-driven PBS dynamics over seconds and minutes, while concomitantly forming Chl *f* over several days (Schmitt et al., 2019, 2020, 2024; Friedrich and Schmitt, 2021). This indicates that structure–function relationships in photosynthetic energy and electron transport in *H. hongdechloris* encompass time scales spanning from 10^−12^ to 10^6^ seconds, covering 18 orders of magnitude in time. The findings on electron tunneling, PBS mobility, and Chl *f* formation serve as paradigmatic examples within a broader spectrum of adaptation processes that operate in photosynthetic organisms.

## 2 Specific results for *H. hongdechloris*

**Figure 1:**
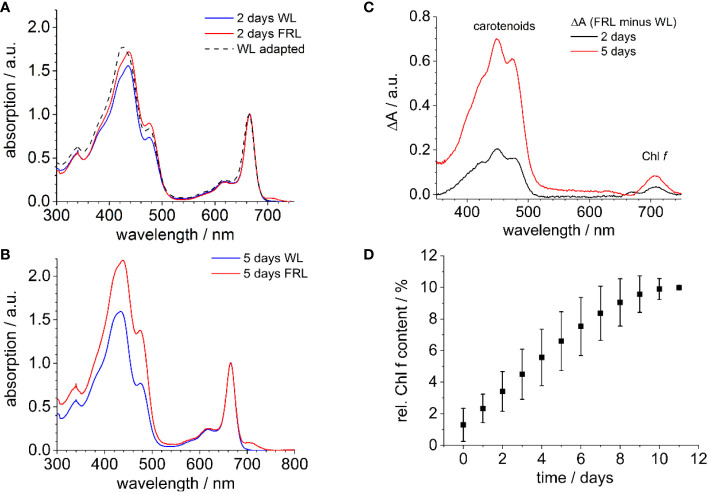
**(A)** Room temperature absorption spectra of methanolic extracts of WL-adapted cells (dashed line). The cells were then further exposed to WL or FRL for 2 days, depicted in blue and red, respectively. **(B)** WL (blue) and FRL (red) illumination following the procedure described in **(A)** for 5 days. The spectra are uniformly normalized at the Chl *a* absorption peak at 665 nm. **(C)** Absorption difference spectra of extracts of *Halomicronema hongdechloris* derived from FRL-grown cells for 2 and 5 days **(A, B)**. **(D)** Relative amplitude of the Q_y_ absorption band of Chl *f* at 707 nm as compared to the Chl *a* absorption at 665 nm after Gaussian fits the bands at 707 nm for Chl *f* and at 665 nm for Chl *a* (n = 5). The figure is reproduced with permission from Schmitt et al. (2019). WL, white light; FRL, far-red light.

**Figure 1** shows the absorption spectra of methanolic extracts of *H. hongdechloris* cells when cultivated under FRL, compared to cells grown under WL as a control (**Figure 1**). The absorption spectra were normalized at 665 nm since the Chl *a* absorption at 665 nm appears as the most stable feature in the chromophore composition during FaRLiP (Schmitt et al., 2024). Notably, within just 2 days of FRL exposure, a new absorption peak not present in WL-grown cells emerged at 707 nm (see **Figure 1A**, Schmitt et al., 2019), a feature that became more pronounced after 5 days (**Figure 1B**). This 707-nm peak was identified as a signature of Chl *f* formation. Additionally, a noticeable rise in absorption in the 400–500-nm region was observed in the FRL-grown samples, which continued to increase over time. The difference spectra in **Figure 1C** closely resemble the absorption characteristics of carotenoids, such as β-carotene, in agreement with previous observations (Chen et al., 2012) and corroborated by analytical data (Li et al., 2014; 2016). This transient increase in carotenoid content may be a common response to shifts in light conditions, particularly within the initial readjustment to a new light environment. When these FRL-adapted cells are transferred back to WL, the Chl *f* content decreases.

To quantify the formation of Chl *f*, the relative presence of Chl *f* at 707 nm was measured using the area under its emission peak compared to the Q_y_ absorption band of Chl *a* at 665 nm, both approximated by a Gaussian function. It was observed that after 12 days under FRL, the absorption of Chl *f* reaches up to 10% relative to Chl *a*, as shown in **Figure 1D**.

The spectra resemble the absorption of chlorophyll molecules but do not show contributions from PBSs because the bilin pigments, which are covalently bound to the PBPs, cannot be extracted by organic solvents (Schmitt et al., 2020; 2024). Therefore, changes due to the redistribution and remodeling of PBSs, as reported in previous studies (Chen et al., 2010, 2012), are not captured in these extracts. During FaRLiP in cyanobacteria, the production of modified PBPs under FRL is well-known (Gan et al., 2014; Gan and Bryant, 2015; Li et al., 2016; Miao et al., 2016; Xu et al., 2016, 2017; Ho et al., 2017a, 2017b; Herrera-Salgado et al., 2018). In *H. hongdechloris*, FRL drives the formation of bicylindrical core structures from APC (Chen et al., 2010, 2012).

The question arises as to how EET can occur from far-red Chl *f* to the reaction center, which at least partially consists of Chl *a*. The mechanisms supporting the charge separation and, finally, water oxidation in PSII are still not fully understood.

**Figure 2:**
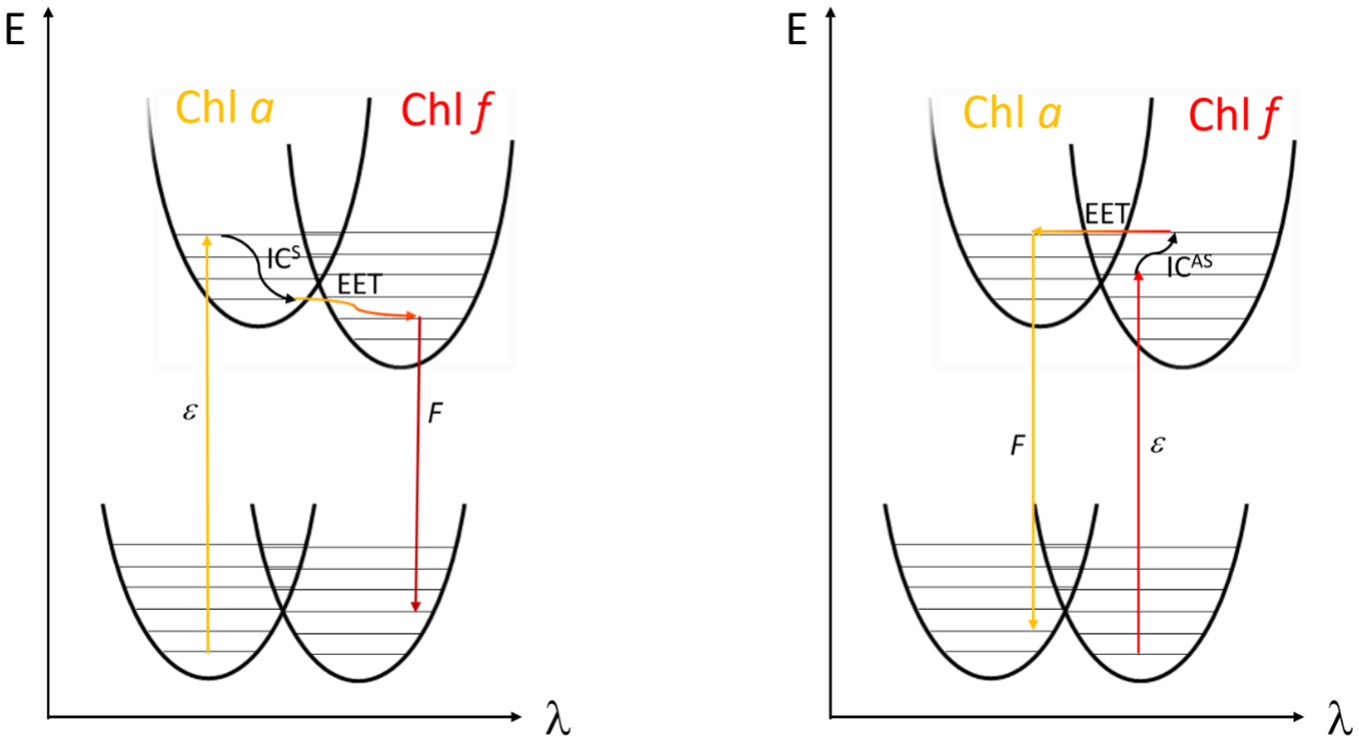
Left side: Fluorescence (F) after Stokes shift compared to absorbed light (ϵ) upon internal conversion by phonon emission (IC^S^) and subsequent excitation energy transfer (EET) from Chl *a* to Chl *f*. Right side: possible EET from Chl *f* to Chl *a* in the form of anti-Stokes internal conversion (IC^AS^) after absorption of phonon energy in the excited state leading to an uptake of energy (adapted with permission from Schmitt, 2020).

**Figure 2** illustrates the mechanism of energy transfer in a coupled Chl *a* (donor) and Chl *f* (acceptor) pair. The left panel depicts the “downhill” energy transfer, in which the electron after light absorption (ϵ) in Chl *a* changes to a lower vibrational level through internal conversion (IC^S^), resulting in a Stokes shift. This leads to energy loss and red-shifted fluorescence (F) from the Chl *f* acceptor. Conversely, the right panel of **Figure 2** depicts a less common scenario. Here, Chl *f* acts as the donor, potentially when being in a vibrationally excited state, enabling an “uphill” transfer to the blue-shifted Chl *a*, which now serves as the acceptor. This process could lead to an anti-Stokes shift in fluorescence due to phonon absorption [anti-Stokes internal conversion (IC^AS^)] followed by EET.

### 2.1 Time-resolved fluorescence spectroscopy to investigate excitation energy and electron transfer

Utilizing time-correlated single-photon counting (TCSPC), spectrally resolved fluorescence decay profiles measured on intact filament bundles of *H. hongdechloris* cells provide direct insights into EET and ET processes. Cells adapted to either WL or FRL (710 ± 10 nm) for 4 days were recorded using 632-nm pulsed excitation at an intensity of 100 W/m^2^ (for details, see Schmitt et al., 2019).

**Figure 3:**
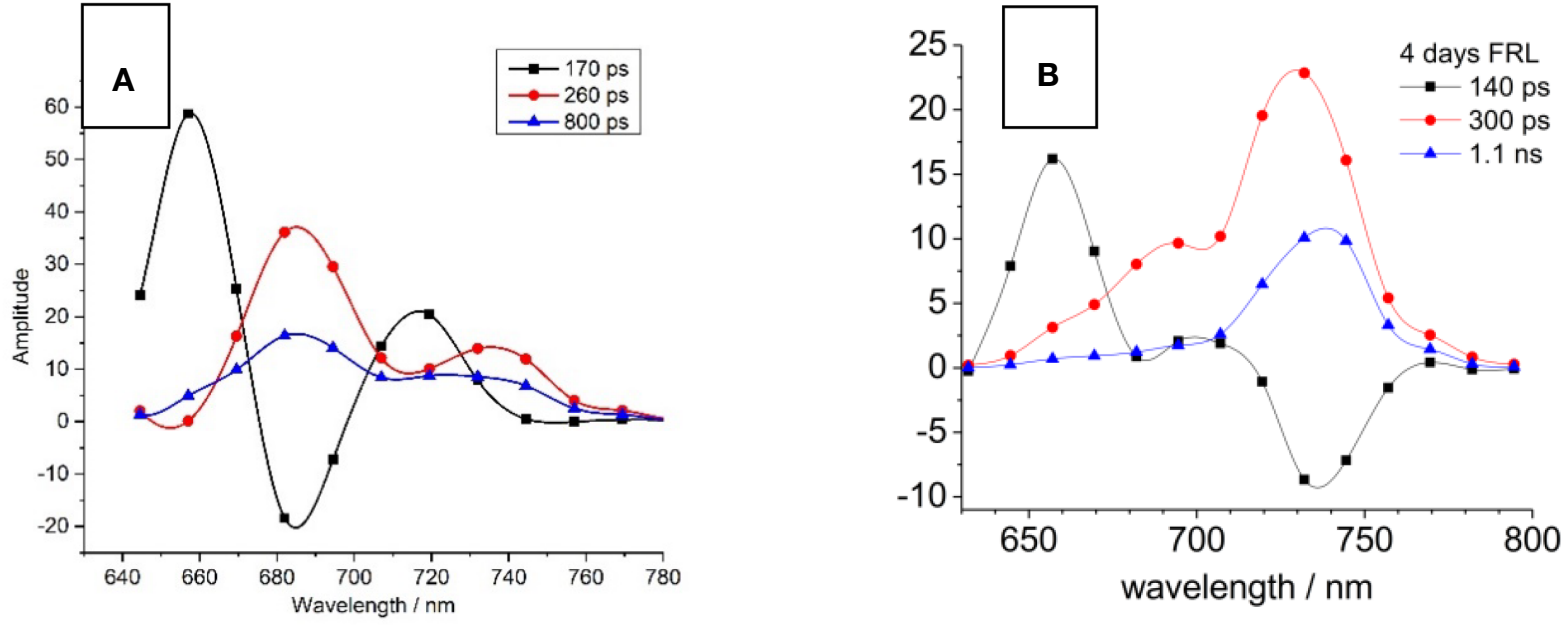
Decay-associated spectra (DAS) of *Halomicronema hongdechloris* cells adapted to white light (WL; A) and far-red light (FRL) for 4 days **(B)**. For both WL- **(A)** and FRL- **(B)** adapted cells, three exponential decay components were identified, with time constants of 140–170 ps (black squares), 260–300 ps (red circles), and 800 ps to 1.1 ns (blue triangles). The data sets are visually connected by cubic spline curves for clarity. Adapted with permission from Schmitt et al. (2019).

The decay-associated spectra (DAS) for both WL- and FRL-adapted cells are shown in **Figure 3**. The DAS for WL-adapted cells reveal the necessity of three exponential decay components for optimal global fitting, with time constants approximating 170 ps (black squares), 260 ps (red circles), and 800 ps (blue triangles) (**Figure 3A**). The fast component (170 ps) exhibits a peak at 660 nm and a negative amplitude (minimum) at 685 nm. Such a negative DAS amplitude represents a fluorescence rise kinetics and describes EET from 660 nm to 685 nm (Schmitt et al., 2019). The two positive components of 260 ps and 800 ps show a maximum at 685 nm where the PD induces charge transfer to Pheo_D1_.

A distinct difference occurs in FRL-adapted cells (**Figure 3B**) since the main emission characterized by the 300 ps (red circles) and 1.1 ns (blue triangles) components is shifted toward 740 nm. Contrastingly, the 140-ps component shows a maximum at 660 nm but a negative DAS amplitude (indicating a fluorescence rise kinetics) at 740 nm, indicating that the EET bridges a much larger spectral distance from PBP absorbing at 660 nm to FR chlorophylls emitting fluorescence at 740 nm. Interestingly, faster EET from PBSs to Chl *f* is observed after FRL acclimation (140 ps, **Figure 3B**, black curve) as compared to the PBS-Chl *a* transfer in WL-adapted samples (170 ps, **Figure 3A**, black curve). This is explained by the fact that PBSs degrade to APC cores during FRL acclimation and that the virtual EET to the RC occurs faster due to the smaller PBS structures.

To rationalize the DAS, EET can be simulated with rate equation models for coupled compartments of PBP, Chl *a*, and Chl *f* (for details, see Schmitt, 2011; Belyaeva et al., 2014).

**Figure 4:**
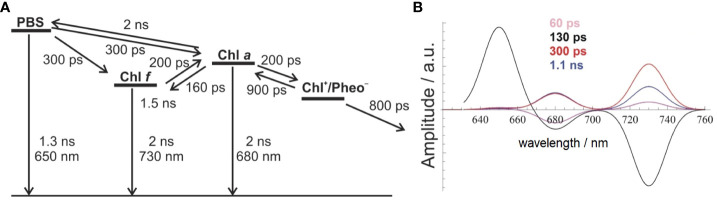
Comparison between experimental and simulated DAS modeled as Gaussian curves with an FWHM of 20 nm **(B)** to account for EET and ET processes in FRL-adapted *Halomicronema hongdechloris* cells upon excitation with 632-nm light, according to the reaction scheme shown in **(A)** The figure is reproduced with permission from Schmitt et al. (2019). DAS, decay-associated spectra; EET, excitation energy transfer; FRL, far-red light.

The best agreement between the measured DAS and the model-based simulations was achieved with the model shown in **Figure 4A** (compare **Figure 4B** to **Figure 3B**). It proposes parallel energy transfer from PBSs to both Chl *a* and Chl *f*. In this model, Chl *f* acts as an intermediary energy storage site in terms of a strongly coupled energy trap, which facilitates uphill energy transfer to Chl *a* and eventually leads to charge separation and the formation of the Chl^+^/Pheo^−^ radical pair with a Chl *a* ^+^ cation, followed by ET to the Q_A_ site. However, the participation of far-red-absorbing pigments like Chl *d* or Chl *f* in charge separation is not excluded. Given the disparity in pool sizes of Chl *a* and Chl *f*, the entropy gain achieved for the transfer from Chl *f* to the 10-fold larger Chl *a* pool supports uphill energy transfer and rationalizes a very short time constant of 200 ps only for this EET step. This mechanism aligns with the Arrhenius–Eyring theory, considering the increased pre-exponential factor due to the entropic term. Further details about the thermodynamics of this model can be found in Schmitt et al. (2019, 2020) and Friedrich and Schmitt (2021). The factor of 10 in the number of Chl *a* as compared to Chl *f* molecules increases the entropy Δ*S* by R·ln(10) per mole (molar gas constant *R* = 8.314 J/K) during EET from Chl *f* to Chl *a*, and the Gibbs free energy of this process is reduced by Δ*S*·*T* = +5.7 kJ/mol at 300 K, therefore accelerating the EET by a factor of 10 (Schmitt et al., 2019).

The model assumes a 200-ps time constant for the Chl *f* to Chl *a* transfer, which closely aligns with the experimental DAS data. The charge separation process is modeled with 200 ps and a subsequent slightly slower charge stabilization (~800 ps) compared to WL-adapted cells. Recombination within the primary radical pair is estimated to be approximately 900 ps. Chl *a* functions as the primary donor in charge separation, providing a redox potential high enough for water splitting. This EET and ET model, as depicted in **Figure 4A**, can be used to simulate DAS (**Figure 4B**), which nicely reflect the experimental DAS (**Figure 3B**). The model (**Figure 4A**) shows EET from PBSs directly to both Chl *a* (at 686 nm) and Chl *f* (at 730 nm), each proceeding with a 300-ps time constant (for the wavelength assignment, see also Schmitt et al., 2019). This model accurately simulates the observed long ~1-ns component at 730 nm and also suggests an additional, minor 60-ps component, which is not resolved in the experiment due to its small amplitude and the width of the instrument response function (IRF; full width at half maximum of 80 ps) (**Figure 4B**).

The depicted reaction scheme in **Figure 4A** presents a minimal model in which Chl *f* acts as an intermediary excitation energy trap, receiving direct excitation from PBSs and being strongly coupled to a larger pool of Chl *a*. However, all Chl *f* species are subsumed as one compartment, and their contribution to charge separation is not discussed. This arrangement, with a low Chl *f* to Chl *a* ratio, aligns with the observed DAS and ensures efficient energy transfer to Chl *a*. The low Chl *f* to Chl *a* ratio is thus optimal for light-harvesting under FRL conditions, although the limited presence of the absorber Chl *f* entails a proportionally lower amount of absorbed FRL energy.

### 2.2 The nature of the primary donor in *H. hongdechloris*

The model shown in **Figure 4** can basically explain the role of Chl *f* in EET, but it does not state the possible involvement of Chl *f* as a PD in the photochemical charge separation. Recent publications from Judd et al. (2020) and Zamzam et al. (2020) suggest a distinct situation of a PD at the Chl_D1_ or P_D2_ position that consists of far-red Chl *d* or Chl *f*, while the secondary donor located at P_D1_ may consist of Chl *a* in the PSII of FRL-adapted *C. thermalis*. In the cryo-EM structure of PSII of FRL-adapted *Synechococcus* sp. PCC 7335 resolved at 2.25Å resolution (Gisriel et al., 2022), one Chl *d* molecule was identified in the Chl_D1_ position, while a Chl *a* was preserved at the P_D1_ position.

There is an absorption band in the range between 470 nm and 500 nm in both WL- and FRL-adapted cells (**Figure 1A**, Schmitt et al., 2024), which may correspond to the Soret band of an FR-absorbing Chl in PSII. The review of the absorption spectra of isolated PSI (Kurashov et al., 2019) and isolated PSII complexes (Judd et al., 2020; Zamzam et al., 2020) indicates that this peak at 470 nm is mainly caused by pigments localized in the PSII. Extensive studies comparing the fluorescence emission upon excitation with 430 nm and 470 nm and the fluorescence excitation spectra for the long-wavelength emission at 730 nm revealed that a peak for 470-nm excitation especially arises in FRL-adapted *H. hongdechloris* cells (Schmitt et al., 2024). In WL-adapted samples, only a minor fraction of far-red fluorescence is excited at 470 nm. In FRL-adapted cells, the 710-nm fluorescence, and especially the 730-nm fluorescence, is preferentially excited between 400 and 470 nm and exhibits a clear peak at 470 nm. Therefore, it is assumed that this peak belongs to the Soret bands of red-shifted Chls, which are formed during FaRLiP (Schmitt et al., 2024).

Even if it cannot be excluded that excitation via carotenoids and subsequent EET to Chl *a* is causing this FR fluorescence to rise, it is clear that the FR emitters in the antenna and/or RC are efficiently excited with 470-nm light. Therefore, the assignment of the 470-nm absorbers as Chl molecules involved in charge separation was tested. For this purpose, low-temperature (10 K) measurements on WL- and FRL-adapted *H. hongdechloris* cells were carried out using pulsed 470-nm excitation (for details, see Schmitt et al., 2024).

**Figure 5:**
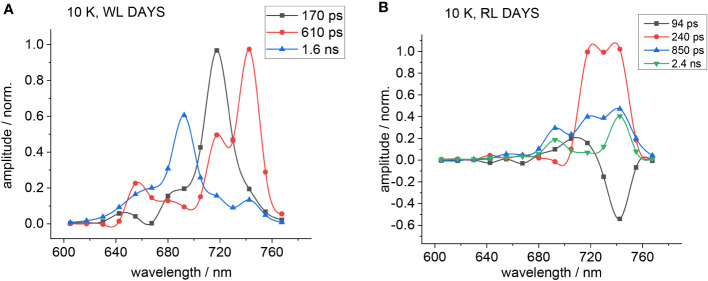
DAYS at 10 K obtained upon excitation with 470 nm for WL- **(A)** or FRL-adapted cells **(B)**. The fluorescence decay curves were approximated with a sum of three exponential components for WL-adapted cells and four components for FRL-adapted cells. The figure is reproduced with permission from Schmitt et al. (2024). DAYS, decay-associated yield spectra; WL, white light; FRL, far-red light.

If amplitudes of fast decay components dominate the spectra and slow time constants exhibit only small amplitudes, it is difficult to judge the contribution of long decay components to the overall DAS. Surprisingly, the fluorescence emission of *H. hongdechloris* after 470-nm excitation appears fast for the FRL-adapted species (see **Figure 5**) (Schmitt et al., 2024). Therefore, the decay-associated yield spectra (DAYS) were used to represent the time-resolved fluorescence data because they allow for better discrimination of long-lived decay components with small amplitudes as compared to DAS.

The determination of the DAYS follows the DAS as described in detail in Schmitt et al. (2024) by multiplying the spectral shape of the fit amplitudes of all decay components in the DAS with the corresponding time constant of the decay component. In contrast to the DAS, the DAYS amplitudes are, therefore, proportional to the overall signal amplitude (registered number of photons) that contributes to a particular component (Schmitt et al., 2024).

**Figure 5** shows the corresponding DAYS for WL-adapted (**Figure 5A**) and FRL-adapted cells (**Figure 5B**) upon excitation with 470 nm at 10 K.

Since PSI fluorescence decays fast even at low temperatures, the fast decay components obtained from DAS analyses result partially from PSI. Therefore, the simulations were conducted with emphasis on obtaining satisfying fits of all decay components except the large fast components at appromimately 720 nm, which are distorted by PSI fluorescence. The DAYS of the FRL-adapted cells (**Figure 5B**) show that the fast decaying fluorescence at 720 nm is small upon FRL acclimation, which underlines the finding that 470-nm light preferentially excites PSII.

The 90-ps (black squares) and 240-ps (red circles) components observed in FRL-adapted samples at 10 K (**Figure 5B**) are coupled between several emitters, indicating that there is still functional and fast EET between the primary donor and the red antenna Chl *f* and that charge separation still occurs even at 10 K with a fast time constant in the regime of 100–200 ps.

In FRL-adapted samples, four decay components are necessary for a satisfactory fit of the fluorescence decays after excitation with 470 nm at 10 K (**Figure 5B**). The fastest component (see black curve in **Figure 5B**) shows a negative amplitude at 740 nm and, therefore, indicates fast EET to Chl *f*. Therefore, it is assumed that the second fast time constant (red curves) represents ET from the primary donor that leads to a fluorescence decay with a 240-ps time constant.

**Figure 6:**
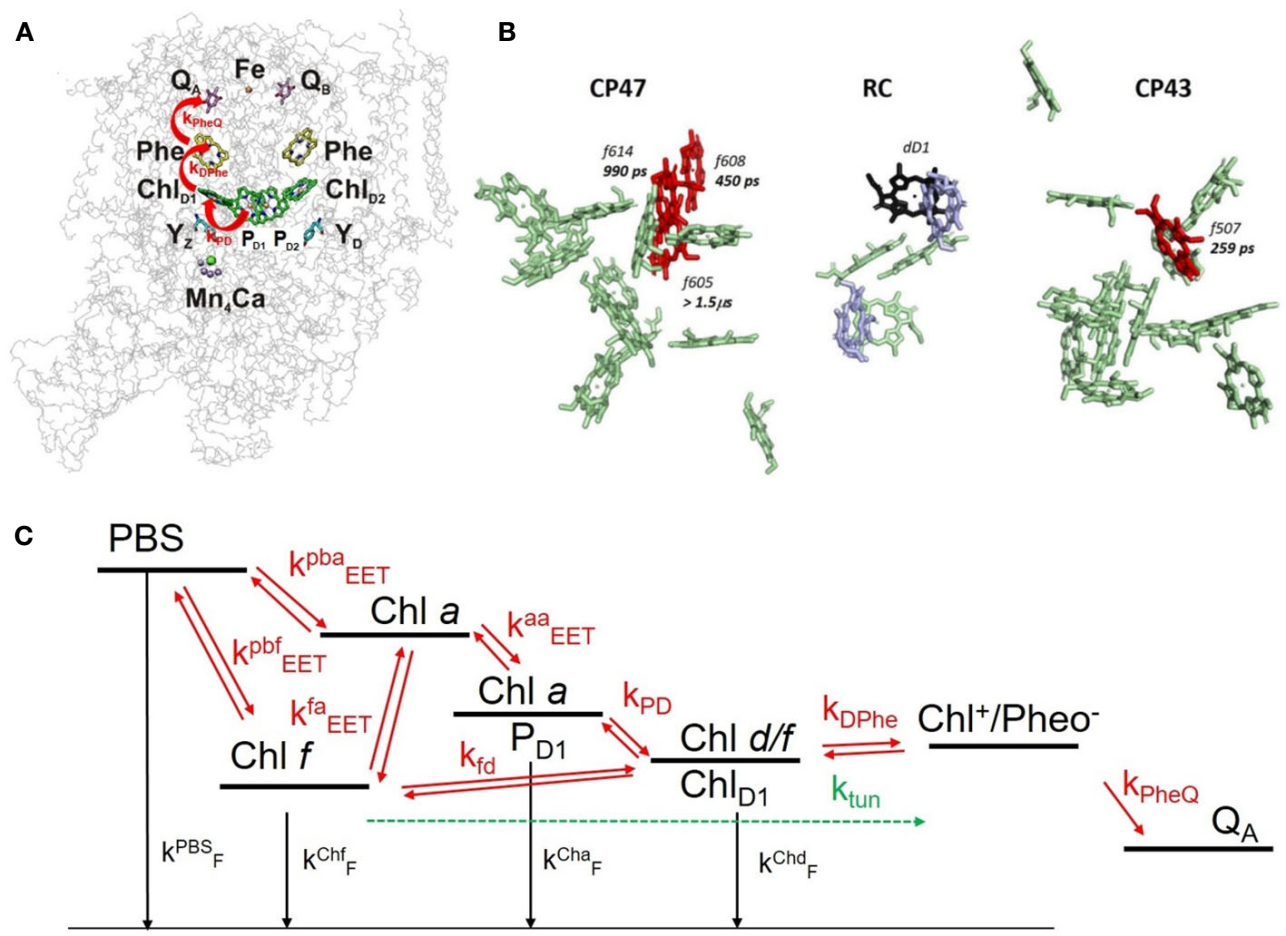
**(A)** 3D structure of the PSII core complex (reprinted with modification with permission from Mamedov et al., 2015). The redox cofactors are shown within the protein backbone: P_D1_ and P_D2_, Chl_D1_, Chl_D2_ as the chlorophyll molecules in the RC; Phe/Pheo, pheophytin, one on D1 and another on D2; Q_A_ (on D2), as a one-electron acceptor plastoquinone; Q_B_ (on D1), as a two-electron acceptor plastoquinone. **(B)** Localization of Chl *f* (red) and Chl *d* (violet) within the PSII of *Synechococcus* sp. PCC 7335 (reprinted with permission from Mascoli et al., 2022). **(C)** Reaction scheme: red arrows indicate transfer processes with rate constants indicated in **Table 1**. The reaction scheme shows the rate constants involved in EET and ET processes occurring in the PSII reaction center of *Halomicronema hongdechloris* as used in the simulation shown in **Figure 7**. The figure is reproduced with permission from Schmitt et al. (2024). PSII, photosystem II; RC, reaction center.

To elucidate the DAYS of WL- and FRL-adapted *H. hongdechloris* cells, a reaction scheme was developed, as described in Schmitt et al. (2024) and visualized in **Figure 6C**. This minimal kinetic model is based on the assignments of Judd et al. (2020); Zamzam et al. (2020), and Gisriel et al., 2022. The DAS are obtained from red antenna molecules and/or the primary donor in the PSII RC since 470 nm preferentially excites the Soret band of red-shifted Chl species (Schmitt et al., 2024). The model is based on **Figure 6A** showing the structural positioning of the chlorins serving as PD and secondary donor. In *C. thermalis*, FR-absorbing Chls are likely located at the Chl_D1_ (PD) and P_D1_ (secondary donor) positions (Nürnberg et al., 2018; Judd et al., 2020; Zamzam et al., 2020). A recent cryo-EM study of far-red light-adapted PSII from *Synechococcus* sp. PCC 7335 at 2.25-Å resolution identified a Chl *d* molecule at the Chl_D1_ position, with Chl *a* preserved at the P_D1_ position under FRL illumination, likely a red-shifted Chl *a* (Judd et al., 2020; Zamzam et al., 2020; Gisriel et al., 2022; Schmitt et al., 2024). It is assumed that four Chl *f* molecules in the CP 43 and CP 47 core antenna complexes and Chl *d* at the Chl_D1_ position are strongly coupled to Chl *a* with similar charge separation dynamics as found in Chl *a*-containing RCs (Gisriel et al., 2022; Mascoli et al., 2022; Viola et al., 2022; Gisriel, 2024, see **Figure 6B**). From the four Chl *f* molecules, three are found in CP 47 with specific single EET transfer rates to the RC of 450 ps (Chl *f* 608), 990 ps (Chl *f* 614), and 1.5 µs (Chl *f* 605) and one in CP 43 with 250-ps EET time to the RC (Chl *f* 507) (see **Figure 6B**). Three of them (Chl *f* 608, Chl *f* 614, and f Chl *f* 507) are well coupled to the RC and support the electron transfer, and one (Chl *f* 605) forms a red trap (Mascoli et al., 2022).

ET in the course of charge separation contains two different terms: i) thermally activated ET proportional to $e^{-\frac{\Delta G}{kT}}$ and ii) electron tunneling (ETUN), which is still visible at 10 K, as it is temperature-independent. Upon cooling to very low temperatures, ETUN remains the only term because the contribution from the temperature-dependent term vanishes (for details, see Schmitt et al., 2024).

The overall transfer *k_ges_* is the sum of both thermally activated ET *k*(*T*) and ETUN (*k*_0_) as given by Equation 1:


(1) $$\begin{array}{l} k_{ges}=k(T)+k_{0}\\ \tau_{ET}=\frac{1}{k_{ges}}=\frac{1}{k(T)+k_{0}} \end{array}$$

and


(2) $$\ln k_{ges}=\ln\left(A_{0}\cdot \exp\left(\frac{-\Delta G}{k_{B}T}\right)+k_{0}\right)$$

At 10 K, *k*(*T*) can be neglected, and $\ln k_{ges}(10\;K)\approx \ln(k_{0})$ (low temperature approximation). At sufficiently high temperatures, ET is determined mainly by the temperature-dependent term *k*(*T*), and the ET rate as given by Equation 2 follows the Arrhenius–Eyring approximation:


$$k(T)=A_{0}\cdot \exp\left(\frac{-\Delta G}{k_{B}T}\right)$$

With the Gibbs–Helmholtz equation ΔG=ΔH−TΔS , it follows that


(3) $$\begin{array}{l} k(T)=A_{0}\cdot \exp\left(\frac{-(\Delta H-T\Delta S)}{k_{B}T}\right)=A_{0}\cdot \exp\left(\frac{\Delta S}{k_{B}}\right)\cdot \exp\left(\frac{-\Delta H}{k_{B}T}\right)\\ \to \ln k(T)=\ln\left(A_{0}\cdot \exp\left(\frac{\Delta S}{k_{B}}\right)\right)-\frac{\Delta H}{k_{B}}\frac{1}{T} \end{array}$$

Equation 3 can be simplified with the following definition:


(4) $$\begin{array}{l} \ln\left(A_{0}\cdot \exp\left(\frac{\Delta S}{k_{B}}\right)\right):=-V\\ \to \ln\frac{1}{k(T)}=\frac{\Delta H}{k_{B}}\frac{1}{T}+V \end{array}$$

This can be simplified so that the slope of ln*k*(*T*) over 1/T equals $\frac{\Delta H}{k_{B}}$ , which is the enthalpy difference between reaction products and educts. The intersection with the y-axis is determined by the expression $V=\ln\left(A_{0}\cdot \exp\left(\frac{\Delta S}{k_{B}}\right)\right)$ and comprises the entropy difference with normalization factor *A*_0_. In this sense, *V* is an effective entropy or degeneration factor for the different numbers of electron donor and acceptor states. The evaluation of Equation 4 on the experimental data (see Schmitt et al., 2024) leads to


$$k_{0}^{WL}={\left(170\text{ ps}\right)}^{-1}\text{ for WL}-\text{adapted samples and}$$

$$k_{0}^{FRL}={\left(240\text{ ps}\right)}^{-1}\text{ for FRL}-\text{adapted samples.}$$

On the basis of **Figure 6C**, the DAS of FRL-adapted *H. hongdechloris* cells at 10 K were simulated. The population dynamics of these states is then modeled with a rate equation system (see Schmitt et al., 2024) as shown in **Figure 6**. In this model, the secondary donor in PSII may consist of a red-shifted Chl *a* at the P_D1_ position, while the PD is an FR Chl (most probably Chl *f*) at the Chl_D1_ position (**Figures 6B, C**). The antenna complexes contain PBSs, Chl *a*, and Chl *f*, which are all functionally coupled by EET (see **Figure 6B**).

**Figure 7:**
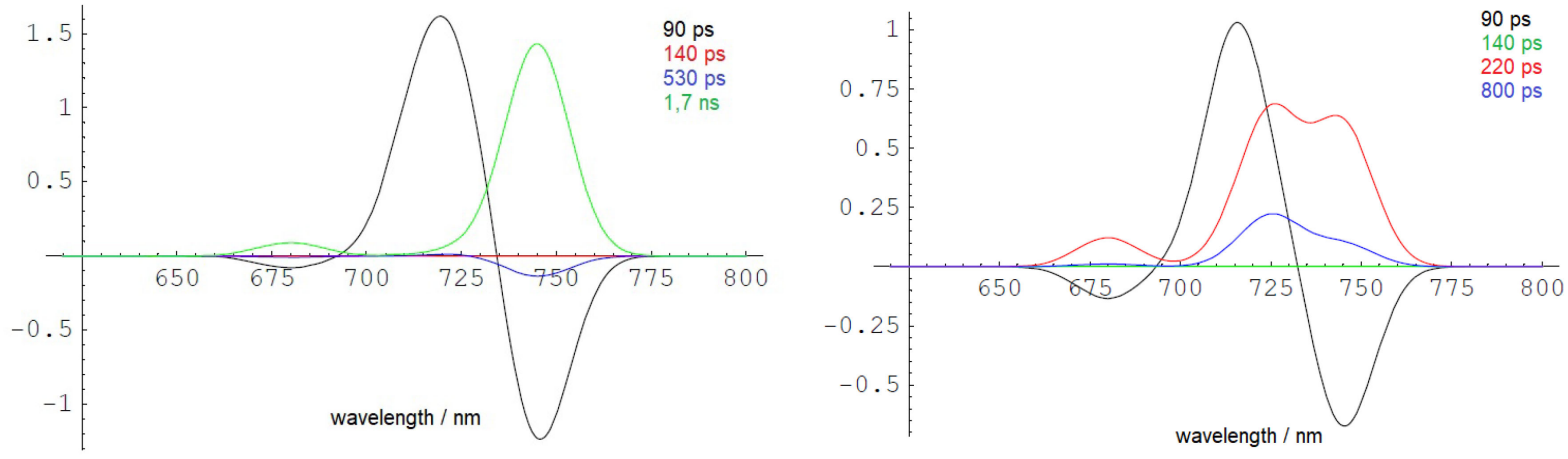
Low-temperature DAS (10 K) simulated with k_ETUN_ = 0 (left side) and k_ETUN_ = 4 ns^−1^ (right side) assuming the EET and ET model shown in **Figure 6**. The DAS are in good agreement with the experimentally observed dynamics, as depicted in the form of DAYS in **Figure 5B**. The figure is reproduced with permission from Schmitt et al. (2024). DAYS, decay-associated yield spectra; EET, excitation energy transfer.

The DAS simulated from the scheme in **Figure 6C** are presented in **Figure 7** with rate constants from **Table 1**. The left side of **Figure 7** displays the outcome of a simulation without incorporating the term for ETUN, while the right side includes an ETUN contribution to the ET rate constant with k_ETUN_ = 4 ns^−1^ (which is written in bold in **Table 1**, corresponding to a 250 ps time constant). This rate is represented by the green arrow indicating **k_ETUN_** in **Figure 6C**. Notably, the correlation between the experimental and simulated DAS, particularly evident on the right side of **Figure 7** compared to **Figure 5B**, demonstrates that a satisfactory match is only obtained if an ETUN process is included. From **Figure 6**, generally, six decay components are expected. However, fast decay components, which cannot be separately resolved as they differ by less than 50 ps in the experimental DAS, were summarized to resemble the shape of a single fast decay component (for details, see Schmitt et al., 2019; Friedrich and Schmitt, 2021).

**Table 1:** Rate constants used to simulate the DAS shown in **Figure 7**.

Rate constant	ns^−1^	Rate constant	ns^−1^
k^pba^_EET_	3.3	k^fa^_-EET_	3
k^aa^_EET_	6	k^aa^_-EET_	0
k^pbf^_EET_	3.3	k^pbf^_-EET_	0
k^fa^_EET_	1	k^fd^_-EET_	3
k^fd^_EET_	4	k_-DPhe_	0.5
k_PD_	20	k^PBS^_F_	0.8
k_DPhe_	5	k^Chf^_F_	0.5
k_PheQ_	1.5	k^Chd^_F_	0.5
k^pba^_-EET_	0	k^Cha^_F_	0.5
**k_ETUN_**	**4**		

Therefore, it can be concluded that charge separation occurs within approximately 200 ps (k_DPhe_) even at 10 K. The recombination probability of the primary radical pair is estimated to be approximately 10% with a 2-ns rate constant. The values are typical for active PSII RCs of organisms performing oxygenic photosynthesis employing Chl *a* at room temperature. Charge stabilization upon ET to Q_A_ (k_PheQ_) fits the experimental data with 670 ps. Thus, EET from Chl *a* to the far-red Chl *a*nd the subsequent ET are efficient even at 10 K.

In simulations excluding the contribution of temperature-independent ETUN (as shown in **Figure 7**, left side), the lowest electronically excited state at 740 nm would rapidly accumulate at 10 K and exhibit a slow decay with an intrinsic lifetime of 1.7 ns, manifesting as an extended fluorescence decay time. However, such a long-lived energy trap is not observed in the cells at 10 K, indicating that the 740-nm fluorescence does not originate from an isolated long-lived trap, even at low temperatures.

### 2.3 Phycobilisome dynamics

**Figure 8:**
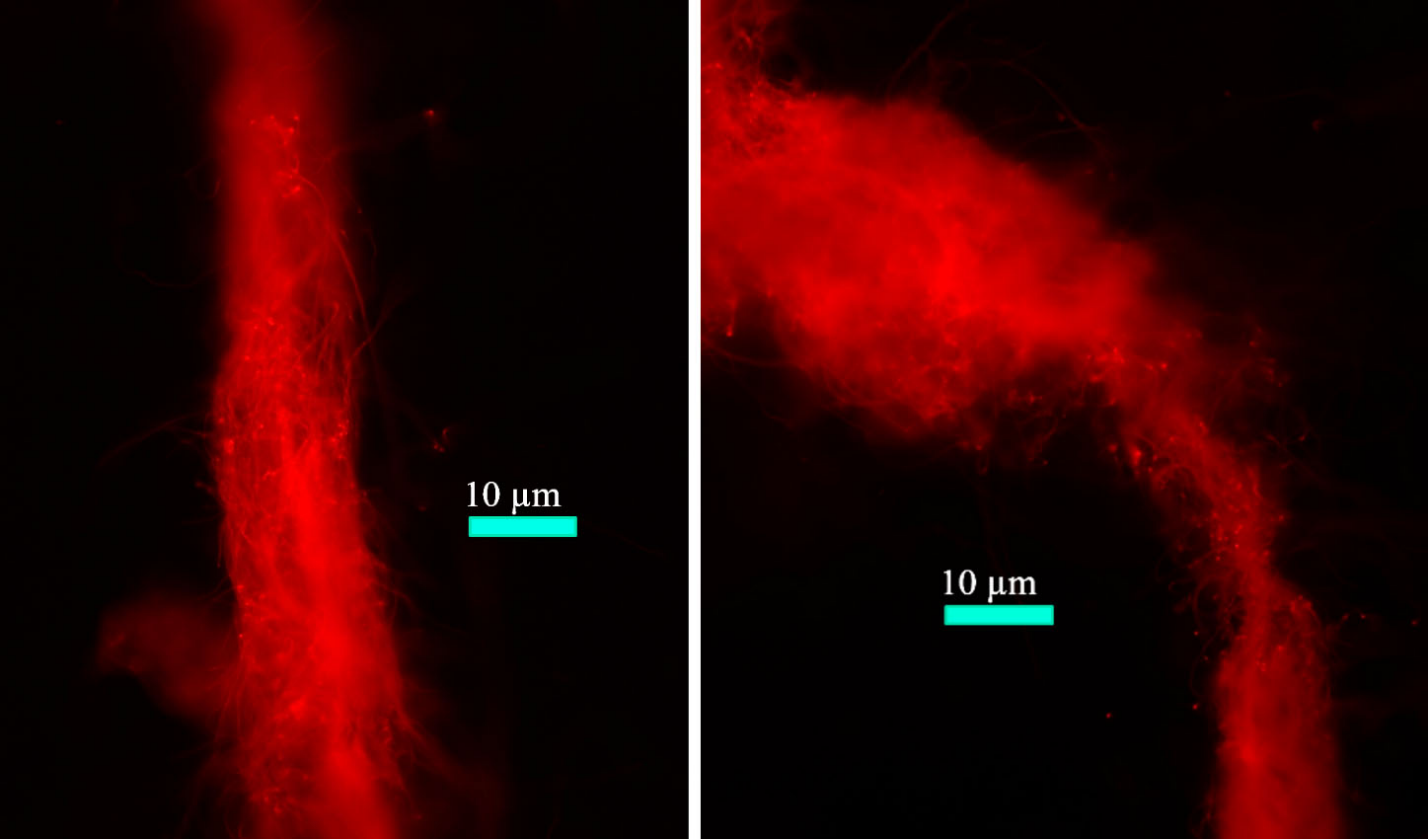
Fluorescence microscopic images of filamentous *Halomicronema hongdechloris* cells. Reprinted with permission from Schmitt et al. (2020).

**Figure 9:**
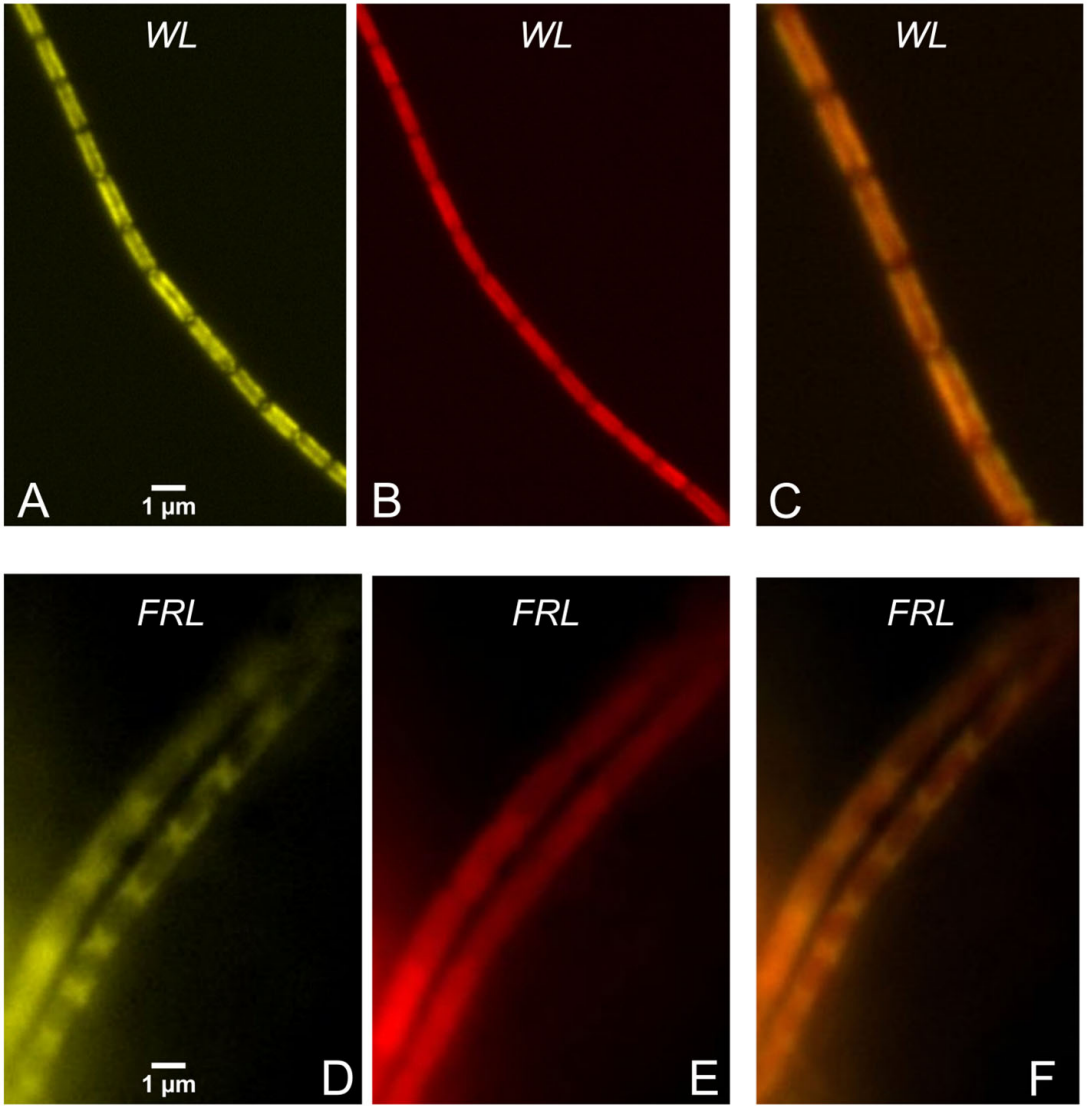
Filaments of *Halomicronema hongdechloris* cells grown under WL **(A–C)** and FRL **(D–F)**. **(A, D)** PBS fluorescence colored in yellow (excitation 405 nm, detection 625–675 nm). **(B, E)** Chl *f* fluorescence (excitation 405 nm, detection > 700 nm) colored in red. **(C, F)** The overlays of the two spectral channels. Note the scale bars in **(A, D)**. Reprinted with permission from Schmitt et al. (2020). WL, white light; FRL, far-red light; PBS, phycobilisome.

*H. hongdechloris* cells typically form filaments (**Figure 8**). When grown under FRL, noticeable changes in the cellular pigment distribution are observed, as depicted in the microscopic images shown in **Figure 9**. These changes have been previously documented and reported in studies by Li et al. (2014, 2016) and Majumder et al. (2017).

**Figure 9** highlights the differences in cellular pigment distribution between WL- and FRL-adapted *H. hongdechloris* cells. In WL-adapted samples, there is colocalization of PBS emission (excitation at 405 nm, detection in the 625–675-nm range) with Chl *f* emission (excitation at 405 nm, detection > 700 nm), as shown in panels A–C. Conversely, in FRL-adapted cells, PBSs are predominantly accumulated at the cell poles, as seen in panel D. In this case, the fluorescence of PBSs does not overlap well with the localization of Chl *f* and other red pigments, as evident in panels D and E.

**Figure 10:**
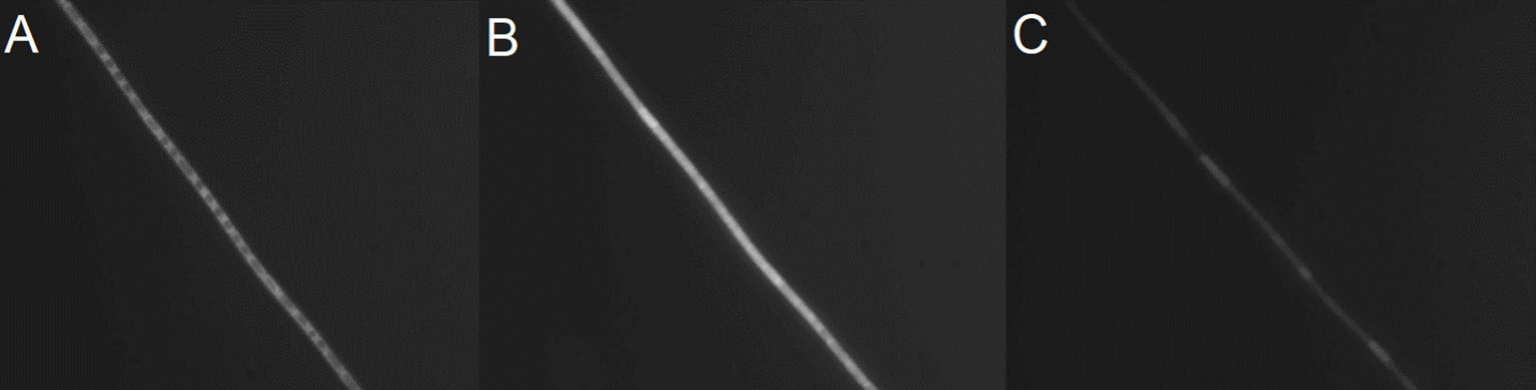
Fluorescence images of filaments of *Halomicronema hongdechloris* cells after 5 days of FRL acclimation **(A)**, excited with 405 nm at an intensity of 500 W/m^2^ for 1 min **(B)** and 2 min **(C)**. Reprinted with permission from Schmitt et al. (2020). FRL, far-red light.

The local redistribution of PBSs after exposure of *H. hongdechloris* filaments previously adapted to FRL to 405 nm measuring light with 500 W/m^2^ intensity is shown in the fluorescence images of **Figure 10**.

**Figure 11:**
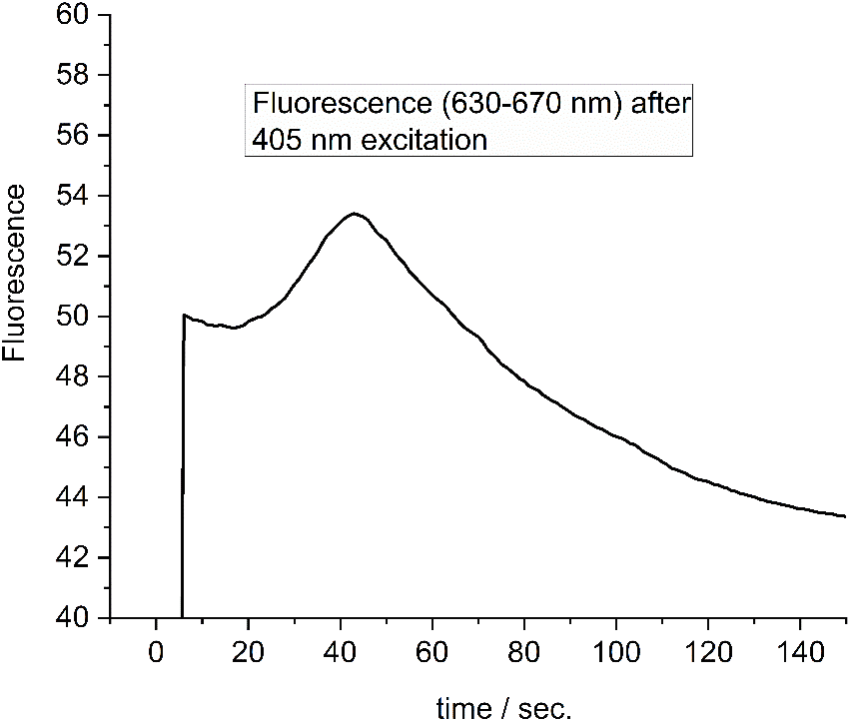
Fluorescence change of a single FRL-adapted cell as shown in **Figures 9**, **10**, **12** upon illumination with 405-nm light. Redrawn with permission from Schmitt et al. (2020). FRL, far-red light.

In **Figure 10A**, the clustering of PBPs between cells was observed upon adaptation to FRL for 5 days, as shown in **Figure 9D**. When these cells were exposed to 405-nm light for 60 seconds (**Figure 10B**), notable changes occurred. Not only does the overall fluorescence intensity increase (see **Figure 11**), but the PBSs also redistribute, leading to a more uniform distribution within each cell, and a subsequent decrease in fluorescence is seen with prolonged 405-nm illumination (**Figure 10C**). Similar transient PBS fluorescence patterns are observed with red (630 nm), blue (405 nm), and white light illumination on similar time scales. However, the dynamics are less pronounced with green light (530 nm), which is predominantly absorbed by the PBSs (Schmitt et al., 2020).

**Figure 12:**
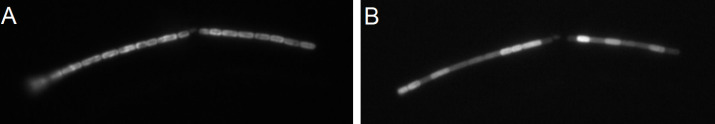
**(A)** FRL-adapted *Halomicronema hongdechloris* cells. **(B)** PBS fluorescence after illumination of FRL-adapted cells with 630-nm light (100 W/m^2^) for 3 min. Reprinted with permission from Schmitt et al. (2020). FRL, far-red light; PBS, phycobilisome.

After 120 seconds of exposure to WL, a significant decrease in fluorescence is observed in certain cells of *H. hongdechloris*, while others retain higher brightness (**Figures 10C**, **12B**). This variation cannot be attributed to photobleaching, which would uniformly affect all cells in the proximity of the light source’s intensity peak. Instead, this differential response in fluorescence intensity is more likely due to intracellular triggers following the initial mobilization of the PBSs under continuous illumination, impacting each cell differently.

The fluorescence intensity of a single FRL-adapted cell during excitation with 405-nm light (500 W/m^2^) is shown in **Figure 11**. A maximum of the fluorescence is reached after approximately 45 seconds. Similar profiles are obtained for all cells (Schmitt et al., 2020).

In **Figure 12B**, the heterogeneous response of individual FRL-adapted *H. hongdechloris* cells to 630-nm light (100 W/m^2^) for 60 seconds is shown. The observed variance suggests the presence of a distinct, cell-specific trigger mechanism. This trigger for the observed rearrangements of the PBSs within individual cells, which we proposed to involve ROS generated by chlorophylls under high light, does not permeate cell membranes but quickly spreads across the whole cell (Schmitt et al., 2020).

Recent research indicates the existence of two distinct types of PSII centers in cells adapted to FRL (Schmitt et al., 2020). These are categorized as type A centers, which contain only Chl *a*, and type B centers, which possess both Chl *a* and Chl *f*. This classification is based on different arrangements of PBSs, comprising either both PC and APC or exclusively APC. This discovery led to the question of where light energy absorbed by PBSs during their transient rearrangement is directed. Consequently, time-resolved fluorescence was used to study the coupling between PBSs and both Chl *a* and Chl *f* within short time intervals after starting the illumination with 632-nm light.

**Figure 13:**
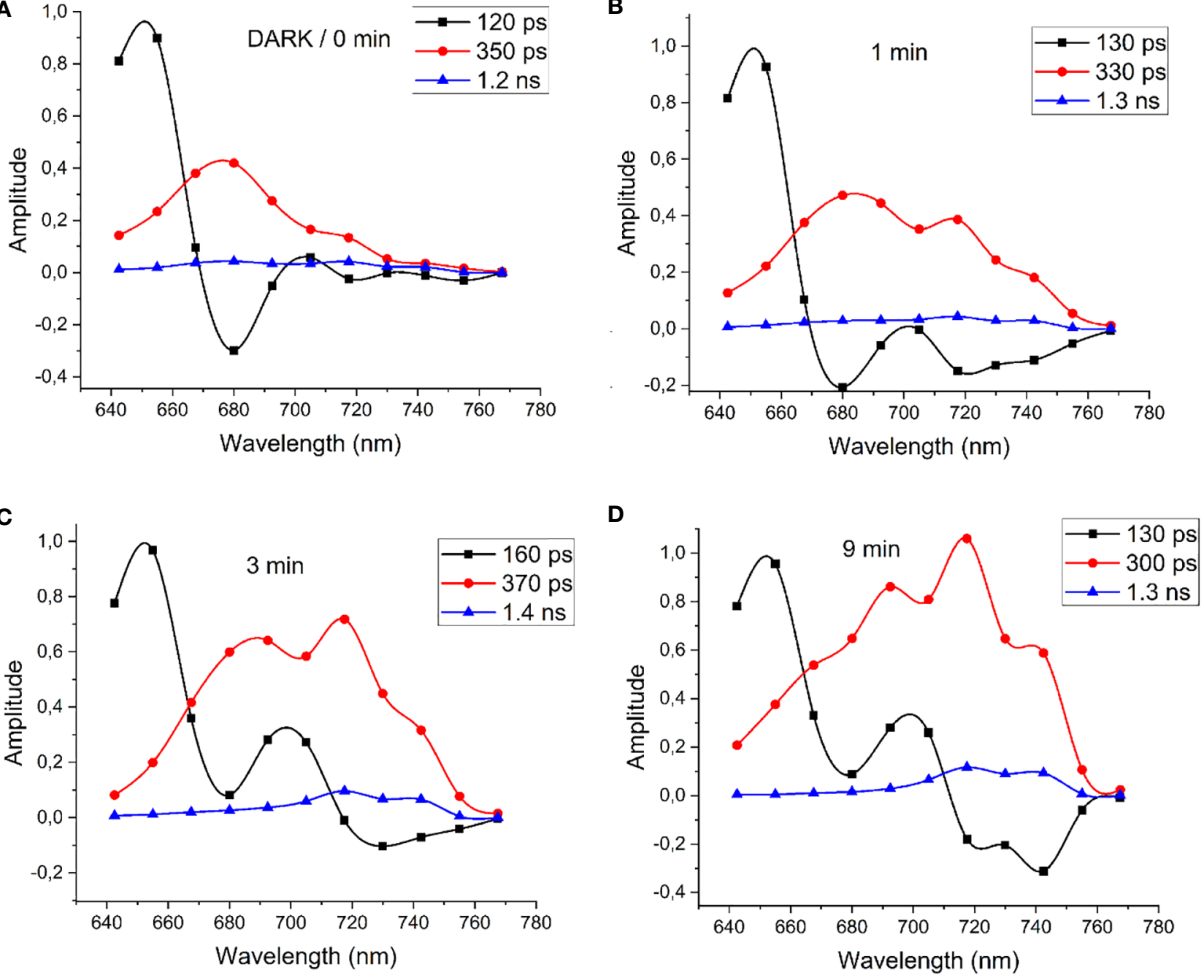
**(A)** DAS of FRL-adapted cells determined immediately after initiating the illumination with 632-nm measurement light of 100 W/m^2^ intensity (“dark”) and after 1 min **(B)**, 3 min **(C)**, and 9 min **(D)** of 632-nm illumination. Modified with permission from Schmitt et al. (2020). DAS, decay-associated spectra; FRL, far-red light.

**Figure 13** shows the DAS of FRL-adapted cells directly after initiating illumination with 632-nm measurement light of 100 W/m^2^ intensity (“dark”, panel A) and during measurement with 632-nm laser irradiation after illumination for 1 min (B), 3 min (C), and 9 min (D).

Upon illuminating FRL-adapted cells with 632-nm light, which strongly excites both Chl *a* and PBSs, notable alterations in the DAS were observed within 10 min. Initially, energy transfer from PBSs to Chl *a* is evident, characterized by a 120-ps component (**Figure 13A**, black squares) peaking at 650 nm and a negative amplitude at 680 nm, with Chl *a* fluorescence decay dominated by a 350-ps component (**Figure 13A**, red circles). The DAS measured directly after FRL acclimation in **Figure 13A** are similar to those of WL-adapted cells, which lack Chl *f*. However, within 1 min of illumination, energy transfer to the long-wavelength region at 715 nm and 740 nm becomes apparent. The time constant for the short EET component between PBSs and Chl *f* gradually increases, reaching a maximum of 160 ps after 3 min, suggesting reduced efficiency in PBS fluorescence quenching. Further illumination leads to pronounced negative fluorescence rise terms at 720 nm and 740 nm with 130-ps time constant indicating that fast EET is re-established from PBSs directly to Chl *f* at 740 nm (**Figure 13D**). Fluorescence at 686 nm, 720 nm, and 740 nm decays with a main component of 300 ps and a small contribution of 1.3 ns (**Figure 13D**, red circles and blue triangles, respectively).

**Figure 14:**
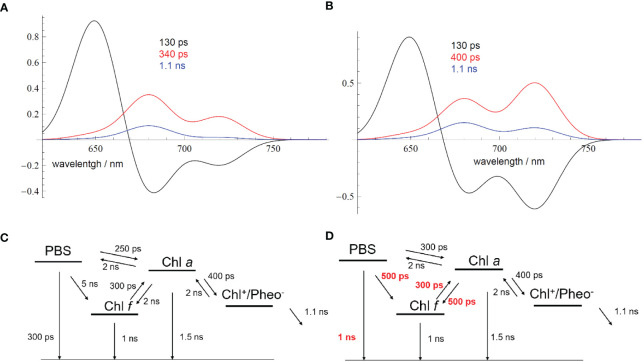
**(A)** Simulated DAS for FRL-adapted samples (**Figure 13A**) directly after starting the measurement according to the EET scheme shown in **(C)**. **(B)** Simulated DAS for FRL-adapted samples after 9-min illumination with 632-nm light of an intensity of 100 W/m^2^ (**Figure 13D**). The changed EET transfer rates due to the ongoing illumination are shown in red in **(D)** Reprinted with permission from Schmitt et al. (2020). DAS, decay-associated spectra; FRL, far-red light; EET, excitation energy transfer.

To simulate the fluorescence dynamics, DAS were calculated (**Figures 14A, B**) according to a simplified reaction scheme as shown in **Figures 14C, D**, which represents a minimal model to describe the PBS dynamics and connectivity between Chl *a* and Chl *f*. As indicated above, two classes of PSII-PBS complexes exist: type A centers, which contain only Chl *a*, and type B centers, which possess both Chl *a* and Chl *f*. PBSs are only bound to type A PSII after FRL acclimation.

The kinetic model presented in **Figure 14** is a clear approximation since the different PSII populations should be independently described according to their own kinetics, with the resulting cellular dynamics being a weighted sum of the two independent dynamics. However, sufficient agreement between the simulated DAS (**Figures 14A, B**) and the experiment (**Figures 13A, D**) was already obtained with a simplified model, which rationalizes the general process of light-induced PBS dynamics.

PBSs degrade under FRL to APC cores only and additionally employ a special red-shifted APC (Li et al., 2016). With 632-nm light, APC cores are strongly excited irrespective of the overall structure of the PBSs. However, the data reveal how FRL accelerates EET from PBSs to Chl *f*, which occurs faster (represented by the 120–140-ps fluorescence component, **Figures 3B** and **13A, D**, black curves) compared to the PBS-Chl *a* transfer in WL-adapted samples (represented as 170-ps fluorescence component, **Figure 3A**, black curve). This is explained by the fact that PBSs degrade to APC cores after FRL acclimation and that the virtual EET to the RC occurs faster due to the smaller PBS structures. It was also observed that PBSs are partially in a quenched state after FRL acclimation and do not transfer excitation energy at all before a fast recovery occurs during short WL illumination (Li et al., 2016; Schmitt et al., 2020).

Therefore, the DAS were simulated (**Figures 14A, B**) with two minimal EET schemes to account for the changes in the fluorescence dynamics (**Figures 14C, D**; see also Schmitt et al., 2020) of FRL-adapted cells at the beginning and after 9 min of illumination with 632-nm laser light. **Figure 14A** describes the resulting DAS in FRL-adapted cells (without preillumination with 632-nm light) upon application of the kinetic scheme in **Figure 14C**, which is in good agreement with the measured DAS shown in **Figure 13A**. **Figure 14B** describes the simulated DAS for the situation after 9 min of illumination with 632-nm light, based on the kinetic scheme in **Figure 14D**, which also agrees with the corresponding experimental DAS in **Figure 13D**. The time constants, which strongly differ upon application of both schemes (**Figures 14C, D**), are marked in red to indicate the main light-induced changes of FRL-adapted cells upon re-illumination with red light.

The DAS simulations and corresponding EET schemes in **Figure 14** reveal that EET from PBSs to Chl *a* in FRL-adapted samples consistently exhibits a time constant of approximately 250–300 ps. This transfer time remains stable during 632-nm illumination, which excites the PBSs (**Figures 14A, B**, noted by the 130–160-ps component, black curve). Also, charge separation at Chl *a* and subsequent charge stabilization at Q_A_ appear similarly unaffected. The dominant decay kinetics in both Chl *a* and Chl *f* regimes occur with approximately 300–350 ps, suggesting charge separation time constants of approximately 400 ps and charge stabilization (ET to Q_A_) with approximately 1 ns.

However, the EET from PBSs to Chl *f* is slow with 5 ns when measured directly after FRL acclimation (see **Figure 14C**), which can be explained by decoupled or even lacking Chl *f* in most of the RCs that receive EET from PBSs. Energy localized on Chl *f* reaches Chl *a* fast within 300 ps, as previously explained by a supporting entropy effect within a large pool of Chl *a* molecules (Schmitt et al., 2019). However, if energy is localized on Chl *a*, only slow transfer to Chl *f* is observed (2-ns time constant, see **Figure 14C**). Since long components mathematically imply that in many centers no or only very slow EET is possible and only small amounts of well-coupled centers exist, the result agrees with the assumption of two types of RCs and that only a few of the RCs are coupled to PBSs. The excited states in PBSs seem to be non-photochemically quenched, which is visible as a 300-ps fluorescence lifetime of the PBSs (see time constants in **Figure 14C**).

**Figure 14D** shows the most prominent changes in the EET pattern induced after 9 min of excitation by 632-nm light on previously FRL-adapted cells. Most evidently, the EET from the PBSs to Chl *f* and also from Chl *a* to Chl *f* accelerates significantly to approximately 500 ps. The intrinsic fluorescence lifetime of the PBSs is prolonged to approximately 1 ns, indicating recovery from the quenched state. The DAS indicate efficient EET from PBSs to Chl *f* (see **Figures 13D**, **14B, D**). The agreement between experimental (**Figures 13A, D**) and simulated DAS (**Figures 14A, B**) is satisfactory within the resolution limits of our measurement.

## 3 Summary of the PBS state transitions

*H. hongdechloris* is a highly adaptive organism that has developed a mechanism to mobilize PBS cores that contain mainly APC and are probably disconnected from Chl *f*-containing reaction centers after acclimation to FRL. Illumination of FRL-adapted samples with blue (405 nm) or red light (630 nm) for several seconds induces mobilization of the PBSs on the time scale of seconds to recouple with Chl *f*-containing PSII and re-establish efficient EET from PBSs to Chl *f*-containing PSII within minutes.

**Figure 15:**
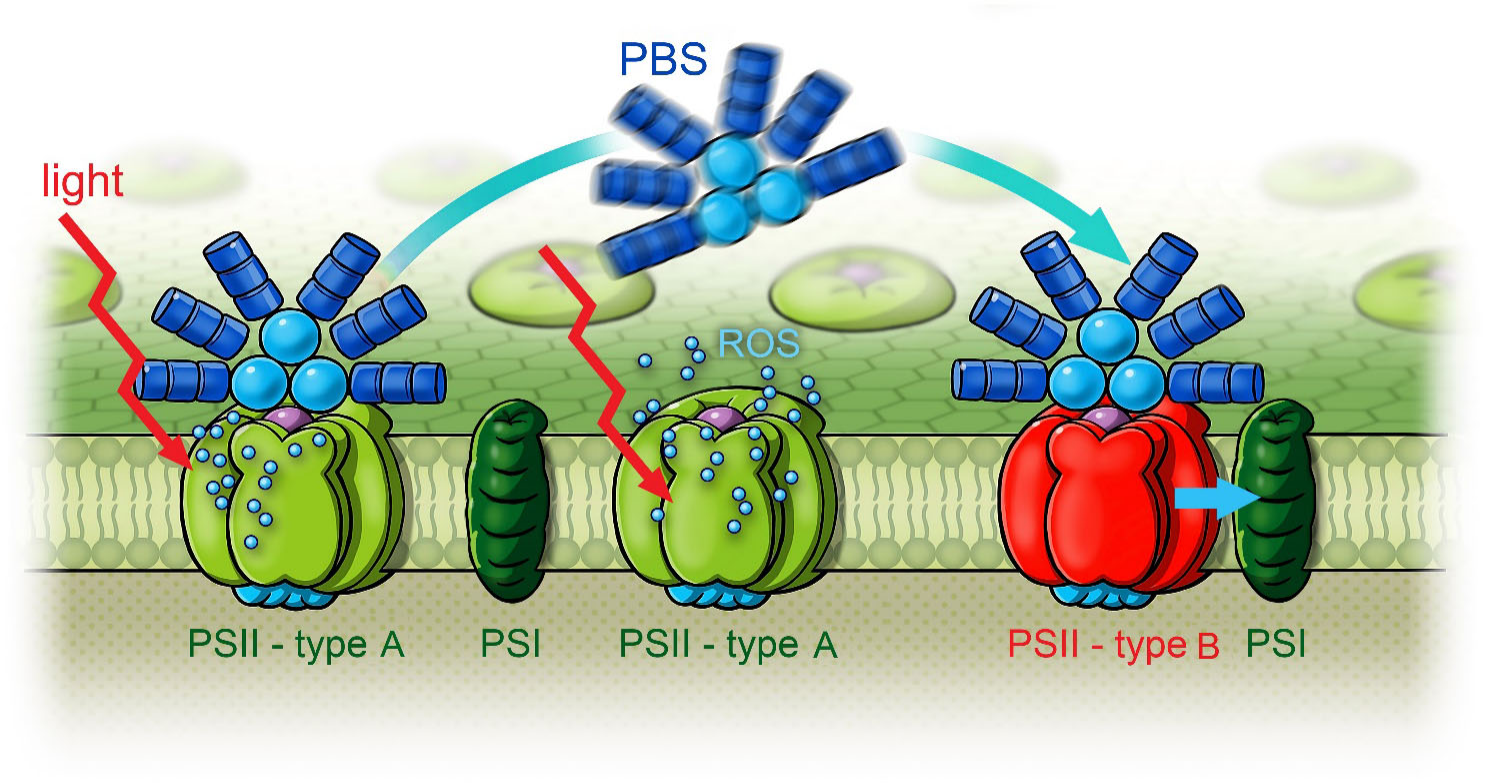
Scheme of white light-induced state transitions from type A PSII to type B PSII in FRL-adapted *Halomicronema hongdechloris* cells. Reprinted with permission from Schmitt et al. (2020). PSII, photosystem II; FRL, far-red light.

To summarize, the results show that after FRL acclimation, the EET from PBSs to Chl *f* and from Chl *a* to Chl *f* is interrupted. EET from PBSs leads to Chl *a* only (see **Figure 14C**). After 3-min exposure to 632-nm light with approximately 100 W/m^2^ (**Figure 13C**), a remodeling of the PBSs becomes visible in the DAS. This occurs with an apparently prolonged lifetime of the fastest component (black squares) of 160 ps instead of 130 ps until finally, after 9 min (**Figure 13D**), a large fraction of the energy absorbed by PBSs is directly transferred to Chl *f*. Energy reaching Chl *a* is quickly equilibrated between Chl *f* and Chl *a*, and the short fluorescence lifetime component of PBSs recovers from 160 ps to 130 ps, indicating efficient EET from PBSs to both Chl *a* and Chl *f*. **Figure 15** illustrates two types of RCs in FRL-adapted cells. After FRL acclimation, PBSs are primarily connected to type A PSII, which contains only Chl *a* and no Chl *f*. Energy absorbed by PBSs is mainly transferred to type A RCs. EET from Chl *a* to Chl *f* is not efficient. Illumination with red, blue, or white light triggers a reconfiguration, re-establishing efficient energy transfer from PBSs to Chl *f* and coupling between Chl *a* and Chl *f*. This process involves a transient decoupling of PBSs from type A PSII and their subsequent association with type B RCs, presumed to be the photochemically active centers formed under FRL, which contain Chl *f*. This dynamic state transition is proposed to significantly enhance photochemical efficiency by equilibrating excitation energy transfer from PBSs between type A and type B RCs as soon as light is available that can be efficiently absorbed by PBSs and drive both type A and type B reaction centers.

It was proposed that ROS formed in type A RCs function as triggers and quickly induce PBS mobilization and redistribution (Schmitt et al., 2020).

*H. hongdechloris* demonstrates remarkable adaptability, enabling it to thrive under FRL conditions. This adaptability is facilitated through CCA, involving the formation of Chl *f* in the PSI and PSII. Additionally, the organism optimizes energy distribution between different forms of PSII complexes. These forms include type B PSII, containing both Chl *f* and Chl *a*, and type A PSII, containing only Chl *a*.

## 4 Summary—reconfiguration of the D1 protein

In FRL-adapted *H. hongdechloris* cells, a minor absorption band at 470 nm can be attributed to the PD of PSII. This donor molecule is most probably a Chl *d* or Chl *f* located at either the Chl_D1_ or P_D2_ position, as suggested by recent studies for other FaRLiP species (Judd et al., 2020; Zamzam et al., 2020; Gisriel et al., 2022; Schmitt et al., 2024; Gisriel, 2024).

In time-resolved fluorescence studies of both WL- and FRL-adapted *H. hongdechloris* cells, a notable charge transfer mechanism was observed at cryogenic temperatures (10 K) upon excitation with 470-nm light. This charge transfer, exhibiting a time constant of 160 ps even at low temperatures, is explained by electron tunneling. In FRL-adapted cells, an additional energy transfer process to 740 nm is evident, suggesting the presence of a long-wavelength energy trap in the antenna system, comprised of Chl *f*. This Chl *f* is closely coupled to the primary donor, facilitating rapid depopulation of its excited states via electron tunneling, which is quantified with a time constant of 240 ps in FRL-adapted cells at 10 K.

In our earlier research (Schmitt et al., 2019), it was established that Chl *f*, formed within 5 days of exposure to FRL, not only functions as an antenna pigment in *H. hongdechloris* but also actively participates in the photochemical processes within the PSII RC. We proposed a model for the composition of the RC formed under FRL conditions. In this model, the primary donor Chl_D1_ is a far-red-absorbing Chl *d* or Chl *f*, while the secondary donor P_D1_, positioned energetically between Chl *a* and Chl *f*, facilitates optimal uphill energy transfer due to its intermediate energy level. This arrangement is particularly efficient for thermal activation from Chl *f* while still maintaining sufficient energy for water splitting.

All these features have been developed by evolution, enabling *H. hongdechloris* to grow in ecological niches enriched in far-red light with varying light conditions. During FRL exposure, the organism accumulates Chl *f* for light absorption and shows fast PBS mobility within seconds to optimally exploit available energy-rich radiation when light conditions suddenly change. These adaptive mechanisms underline the flexibility and innovative capacity of photosynthetic organisms, which persistently improve their strategies for the effective use of solar energy.

Adaptations in PSII are characterized by alterations in the D1 and D2 proteins, which are critical for the PSII reaction center’s function. The amino acid sequence changes observed in these proteins from FRL-adapted organisms suggest a tailored binding environment for Chl *f*, facilitating its integration into the PSII core (Ho et al., 2016; Elias et al., 2024). These structural adaptations in PSII contribute to improved charge separation efficiency under FRL. This is supported by electron tunneling and the stabilization of the charge-separated state, ensuring effective electron transfer even at lower energy levels provided by FRL (Viola et al., 2022; Elias et al., 2024).

## 5 Outlook and applications

The elucidation of these specific structural and functional modifications in PSI and PSII after FaRLiP offers valuable insights into the mechanisms underlying FRL acclimation in photosynthesis. For researchers focusing on FRL acclimation, these findings provide a critical foundation for understanding the strategies that enable photosynthetic organisms to exploit extended light spectra and adapt to varying environmental conditions. This could also open up opportunities for biotechnological approaches aimed at increasing the productivity of crops by expanding the usable spectrum of light for photosynthesis.

Incorporating the knowledge of how organisms like *H. hongdechloris* adapt to FRL conditions into broader research and applications could open up several innovative avenues across various fields, from agriculture to synthetic biology. The key benefits and opportunities for exploration based on this capability may be as follows:

**Agricultural applications**: Understanding how cyanobacteria adapt to and thrive under FRL can help develop crops that use light more efficiently, especially in densely planted fields where lower leaves successively receive light from the far-red spectrum. By manipulating or mimicking the mechanisms in crop plants that enable the synthesis and targeted integration of Chl *f*, it may be possible to enhance their photosynthetic efficiency and, consequently, their growth and productivity. This could be especially beneficial in algae farms, in regions with limited sunlight or vertical farming settings.

**Biomimetic solar energy capture**: The unique adaptations of *H. hongdechloris* to utilize FRL through Chl *f* can inspire the design of advanced photovoltaic materials that mimic these biological processes. Such bio-inspired solar panels could potentially capture a broader spectrum of solar radiation, including the far-red and near-infrared wavelengths.

**Climate change mitigation**: Enhancing the photosynthetic efficiency of plants and cyanobacteria could increase carbon capture. By engineering terrestrial plants or aquatic bio-systems that can utilize the light of the available solar spectrum more efficiently, these systems could fix more CO_2_, contributing to the mitigation of climate change.

**Synthetic Biology and biotechnology**: The enzymes and genetic pathways involved in Chl *f* synthesis and its integration into photosystems represent exciting targets for synthetic biology. Engineering of organisms expressing these traits could lead to the development of novel bio-production systems that operate more efficiently in algae farms or under light conditions not suitable for traditional agricultural methods.

**Ecological and environmental studies**: A deeper understanding of how different light spectra influence biodiversity and ecosystem productivity could have significant implications for conservation biology and environmental management. This could be particularly relevant in shaded environments such as forest understory and deep-water systems where light quality significantly impacts the local biota.

**Evolutionary biology insights**: Exploring the evolutionary pathways that have allowed certain cyanobacteria to harness FRL could provide insights into the mechanisms shaping the evolution of photosynthetic adaptations. This could help in reconstructing the evolutionary history of photosynthesis and offers clues about early life forms on Earth and, potentially, on other planets.

These ideas provide a roadmap for future research and application opportunities and highlight the interdisciplinary potential of current findings on FRL acclimation in photosynthetic organisms.

## Author contributions

FS: Conceptualization, Data curation, Formal analysis, Investigation, Methodology, Project administration, Supervision, Validation, Visualization, Writing – original draft, Writing – review & editing. TF: Conceptualization, Funding acquisition, Methodology, Project administration, Resources, Supervision, Writing – review & editing.
